# Dietary amino acids and anthropometric indices: Tehran Lipid and Glucose Study

**DOI:** 10.20945/2359-3997000000646

**Published:** 2023-06-19

**Authors:** Farshad Teymoori, Golaleh Asghari, Sanaz Hoseinpour, Sajjad Roosta, Maryam Bordbar, Parvin Mirmiran, Narges Sarbazi, Fereidoun Azizi

**Affiliations:** 1 Shahid Beheshti University of Medical Sciences Research Institute for Endocrine Sciences Nutrition and Endocrine Research Center Tehran Iran Nutrition and Endocrine Research Center, Research Institute for Endocrine Sciences, Shahid Beheshti University of Medical Sciences, Tehran, Iran; 2 Iran University of Medical Sciences School of Public Health Department of Nutrition Tehran Iran Department of Nutrition, School of Public Health, Iran University of Medical Sciences, Tehran, Iran; 3 Islamic Azad University Department of Nutrition, Science and Research Branch Tehran Iran Department of Nutrition, Science and Research Branch, Islamic Azad University, Tehran, Iran; 4 Lorestan University of Medical Sciences School of Health and Nutrition Student Research Committee Khorramabad Iran Student Research Committee, School of Health and Nutrition, Lorestan University of Medical Sciences, Khorramabad, Iran; 5 Shahid Beheshti University of Medical Sciences Endocrine Research Center Research Institute for Endocrine Sciences Tehran Iran Endocrine Research Center, Research Institute for Endocrine Sciences, Shahid Beheshti University of Medical Sciences, Tehran, Iran

**Keywords:** Amino acid, weight change, waist circumference, body adiposity index, body mass index, obesity

## Abstract

**Objective:**

Recent studies investigated the role of amino acids (AAs) in weight management. We aimed to determine the association between AAs and three-year change of anthropometric indices and incident obesity.

**Materials and methods:**

Height, weight, hip, and waist circumference (WC) were collected at baseline and follow up. Three-year changes in anthropometric indices and obesity incident according to body mass index (BMI) (overweight & obesity) and WC cutoffs (obesity-WC) were ascertained. Dietary intakes of AAs were collected at baseline, using a food frequency questionnaire. Data analyses were conducted on 4976 adult participants and two subsamples, including 1,570 and 2,918 subjects, for assessing the AAs relationship with 3-year changes on anthropometric indices and obesity incident.

**Results:**

Lysine and aspartic acid were positively associated with higher weight change, whereas acidic AAs, cysteine, and glutamic acid showed a negative correlation with weight change. Furthermore, a weak positive correlation was shown for alkaline AAs, lysine, and valine with WC; however, acidic AAs, tryptophan, cysteine, and glutamic acid were negatively associated with WC. Aromatic and acidic AAs also demonstrated a weak negative relation with changes in BAI. Phenylalanine and Aromatic AAs showed a negative association with overweight &obesity incidence adjusting for potential confounders. Each quartile increases the dietary lysine, arginine, alanine, methionine, aspartic acid, and alkaline AAs related to a greater risk of obesity-WC, while tryptophan, glutamic acid, proline, and acidic AAs associated with lower obesity-WC risk.

**Conclusion:**

Our results suggested that certain dietary AAs may potentially change anthropometric indices and risk of obesity incident.

## INTRODUCTION

Excess weight is a major world health problem contributing to many chronic diseases such as diabetes mellitus and cardiovascular diseases (1). Obesity is a multifactorial disease, and some of its recognized related factors are diet, genetic, sedentary lifestyle, and metabolic disorders (2). Dietary factors have an important role in weight change, and the association between weight gain and intake of macronutrients has been extensively investigated (3). Protein has more satiating and thermogenic effects than carbohydrates and fat; therefore, it might have preventive effects on excess weight (4). While some cross-sectional studies showed that high protein diets are associated with weight loss and waist circumference (WC) reduction (5,6), findings of cohort and interventional studies are inconsistent; some of these studies found a positive correlation between higher protein intake and both weight and WC reduction (7,8), some others showed no association (9). The metabolic actions of dietary proteins were attributed to their amino acid components and food sources (10). AAs are dietary components with several actions such as being utilized as body fuel or in neurotransmitter activities. In the past decade, AAs received more attention regarding their possible roles in different metabolic conditions such as obesity, insulin resistance (IR), diabetes, hypertension, and cardiovascular disease (CVD) (11). Studies indicated changes in AAs plasma levels in obese individuals (12); however, the association of usual dietary intake of AAs with body weight and obesity indices was investigated in few cross-sectional studies (13). In the study by Li and cols., higher dietary intake of branched-chain AAs (BCAAs) was associated with a lower risk of obesity (14). Okekunle and cols. reported that a total and 13 individual AAs were inversely associated with obesity risk (13). In addition, in Chinese adults, sulfuric AAs (SAAs) and histidine showed a respectively positive and negative relationship with obesity (15). Regarding the daily consumption of AAs and the worldwide epidemic of obesity, it seems that indicating the possible relationship between AAs and weight is essential. Therefore, we aimed to investigate the association between individuals and AA groups with 3-year changes of anthropometric indices including body weight, WC, and body adiposity index (BAI), and obesity incident as a prospective study design among participants of the Tehran Lipid and Glucose Study (TLGS).

## MATERIALS AND METHODS

### Participants

This study was conducted within the framework of the TLGS, a prospective study initiated in 1999, whit the aim to determine the prevalence of non-communicable disease risk factors among Tehran’s urban population (16), with participants being monitored every three years; the baseline survey was a cross-sectional study (1999-2001), and surveys II (2002-2005), III (2006- 2008), and IV (2009-2011) were prospective follow-up surveys.

**Figure 1:**
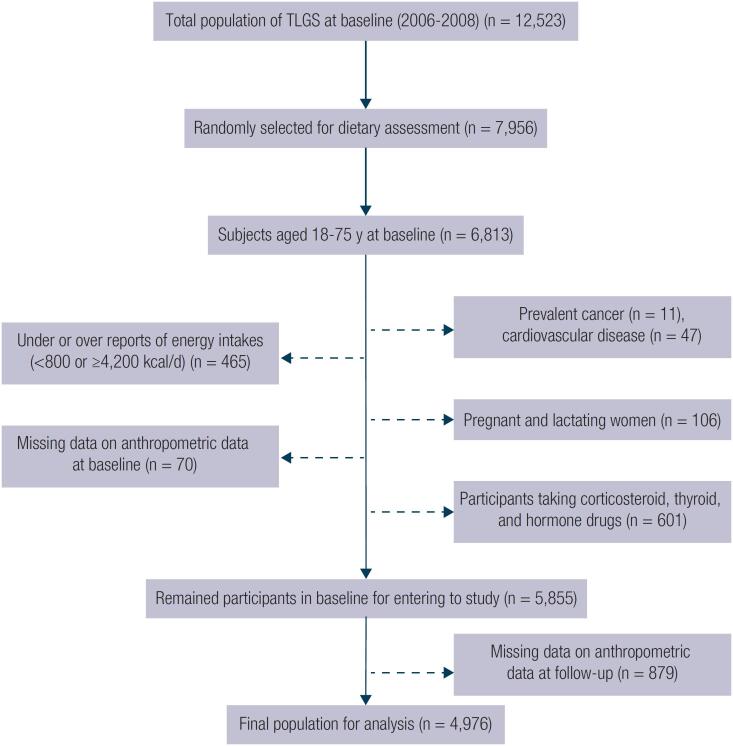
The flow chart of the study participants and follow-up

In the fourth survey of the TLGS (2009-2011), from 12,823 participants, 7,956 were randomly selected and agreed to complete a dietary assessment. For the present study, from 6,813 individuals aged 18 to 75 years, those with prevalent cancer (n = 11), cardiovascular disease (n = 47), pregnant and lactating women (n = 106), participants taking corticosteroid, thyroid, and hormone drugs (n = 601), and participants with under- or over-reported dietary intakes (less than 800 kcal/d or more than 4,200 kcal/d, respectively), (n = 465) were excluded. For assessing anthropometric changes, after exclusion of those without data on weight and waist (n = 70), 5,855 participants entered the study. After excluding the subjects whose anthropometric data were not collected in the follow up (n = 879), 4,976 remained for the final analysis of 3-year changes of anthropometric data including BMI, WC, and BAI (follow up rate: 84.9%) (Figure 1). Some individuals fell into more than one category.

We also conducted two separate analyses to investigate the association of dietary AAs and the risk of overweight and obesity incidence once defined based on BMI cut off (overweight & obesity) and once again based on WC for Iranian population cutoff (obesity-WC) after 3-years of follow up. So that we excluded participants who were overweight & obese based on BMI cut off (n = 3,406) and who were obese based on WC cutoff (n = 2,060), and final analysis were conducted on 1,570 and 2,918 participants for assessing the dietary AAs relationship with the risk of incidence of overweight & obesity and obesity-WC, respectively.

Written informed consent was obtained from all participants, and the study protocol was reviewed and approved by the ethics research council of the Research Institute for Endocrine Sciences, Shahid Beheshti University of Medical Sciences by ethic code IR.SBMU.ENDOCRINE.REC.1398.062.

### Dietary intake assessment

Dietary intakes were assessed using a valid and reliable semi-quantitative food frequency questionnaire (FFQ). The reliability and validity of the FFQ have been previously reported (17). During the previous year, consuming each food item’s frequency on a daily, weekly, or monthly basis was collected during a face-to-face interview by trained and experienced dieticians. Portion sizes of consumed foods, reported in household measures, were then converted to grams. Using the United States Department of Agriculture (USDA) food composition table (FCT), energy and nutrient content were computed. For local food items that were not available in USDA FCT, the Iranian FCT (18) was used. Data on AAs was calculated using USDA (USDA National Nutrient Database for Standard Reference, Release 28) FCT of 2015 (http://www.ars.usda.gov/ba/bhnrc/ndl), which is based on the chemical analysis of AA composition of over 5000 food items from all food groups. Dietary intakes during the fourth phase (2008-2011) of TLGS were considered as dietary intake exposure at baseline.

### Physical activity assessment

Estimating physical activity levels was done using the Modifiable Activity Questionnaire, which was formerly adjusted and validated among Iranians (19). Participants were asked to report the frequency and time spent on activities of light, moderate, hard, and very hard intensity during the past 12 months according to a list of everyday activities of daily life; physical activity levels were represented as metabolic equivalent hours per week (MET-h/wk).

### Anthropometric measurement

Information on age, sex, medical history, medication use, and participants’ smoking habits were collected by trained interviewers using pretested questionnaires. Anthropometric measures, including weight, height, and waist circumference (WC) were measured, and body mass index (BMI) was calculated. Weight was measured with an accuracy of up to 100 g using a SECA digital weighing scale (Seca 707; Seca Corporation, Hanover, Maryland; range, 0.1-150 kg) with subjects wearing minimal clothing. Height measurement was performed while participants were barefoot and in a standing position, with shoulders in normal alignment, using a stadiometer with an accuracy of 1 mm. Body mass index was calculated as weight in kilograms, divided by height in meters squared. Waist circumference was measured to the nearest 0.1 cm using an unstretched shape tape meter, at the umbilicus level, over light clothing, without any pressure on the body surface.

Body adiposity index (BAI): BAI calculated as (hip circumference)/((height)^1.5^) − 18) which is an alternative parameter for calculation of %body fat and can be used to reflect % body fat for adult men and women in different ethnicities (20).

Overweight and obesity (overweight & obesity): Defined according to the World Health Organization (WHO) standards, which considered the BMI ≥ 25 kg/m^2^ as overweight.

Obesity based on WC for the Iranian population (obesity-WC): We defined obesity based on the WC cutoff points 99.5 cm for men and 94.25 cm for women, which has been corresponded to BMI values of ≥ 30 kg/m^2^ among the Iranian population (21).

### Statistical analysis

Statistical Package for Social Sciences (version 15.0; SPSS Inc, Chicago IL) was used for data analyses. Assessing the normality of variables was done using Kolmogorov-Smirnov and histogram charts. Baseline characteristics of subjects were expressed as mean ± SD or median (25-75 interquartile range) for quantitative variables and percentage and number for qualitative variables. AAs intake was reported as a percentage of total protein intake. The 3-year changes in body weight, WC, and BIA were ascertained and dietary individual and group AAs were categorized based on quartiles.

Multiple linear regression analysis was conducted with body weight, WC, and BIA change values as dependent variables and quartiles of AAs (% of total protein) as an independent continuous variable. Beta coefficient (unstandardized) and their respective confidence intervals 95% (95% CI) were reported. Multiple logistic regression analysis was used for assessing the association of dietary AAs and incidence of overweightness & obesity or obesity-WC after 3-years of follow up. Overweightness & obesity or obesity-WC incidence as a dichotomous variable was entered as a dependent variable. Each quartile increase of dietary AAs in the study baseline was entered for analysis as independent variables and odds ratio (OR) with 95% CI reported.

All analyses were adjusted for potential confounders, including age, sex, BMI, physical activity, smoking, daily intake of energy, total fat, and fiber, besides anthropometric factors baseline values. Two-tailed P values were reported, and P < 0.05 were considered statistically significant.

## RESULTS

Characteristics of all participants, males, and females are shown in Table 1. The mean ± SD age and BMI of participants (44.8% males) were 41.2 ± 13.4 and 27.3 ± 4.8, respectively. The yearly median and interquartile range (IQR) of body weight, WC, and BAI changes of the total study population were 0.33 (-0.33, 1.33) kg/y, 0.00 (-1.00, 1.33) cm/y, and -0.12 (-0.44, 0.30), respectively.

**Table 1:** Baseline characteristics of participants of the Tehran Lipid and Glucose Study (2008-2011)

Characteristics	Total (n = 4,976)	Males (n = 2,230)	Females (n = 2,746)	P-value
**Baseline**				
Age (year)	41.2 ± 13.4	42.0 ± 13.9	40.5 ± 13.0	<0.001
Body mass index (kg/m^2^)	27.3 ± 4.8	26.9 ± 4.1	27.7 ± 5.2	<0.001
Smoking (%)	532 (10.7)	461 (20.7)	71 (2.6)	<0.001
Physical activity (MET/h/week)	65.0 (32.7, 98.3)	86.7 (55.8, 117.8)	49.1 (25.9, 76.8)	<0.001
Weight (kg)	73.2 ± 14.4	79.9 ± 13.5	67.7 ± 12.6	<0.001
Waist circumference (cm)	92.4 ± 12.1	95.5 ± 11.0	89.8 ± 12.3	<0.001
Body adiposity index	30.2 ± 5.7	26.2 ± 3.5	33.5 ± 5.1	<0.001
**Follow-up**				
Weight (kg)	74.7 ± 14.8	81.5 ± 14.2	69.1 ± 12.8	<0.001
Weight change (kg/y)	0.33 (-0.33, 1.33)	1.0 (-1.0, 4.0)	1.0 (-1.0, 4.0)	0.235
Waist circumference (cm)	93.0 ± 12.1	96.2 ± 10.7	90.3 ± 12.5	<0.001
Waist circumference change (cm/y)	0.00 (-1.00, 1.33)	1.0 (-2.0, 3.0)	0.0 (-3.0, 4.0)	0.270
Body adiposity index	30.1 ± 6.1	25.7 ± 3.5	33.6 ± 5.5	<0.001
Body adiposity index change	-0.12 (-0.44, 0.30)	-0.44 (-1.35, 0.44)	0.0 (-1.12, 1.34)	<0.001

Data represented as mean ±SD, or median (interquartile range), or number and percent.

Dietary intakes of participants are presented in Table 2. Generally, participants have a usual diet with macronutrient composition of total energy, including 56.7% carbohydrate, 28.9% fat and 13.3% protein. As a percentage of total protein, Glutamic and aspartic acid were the most predominant dietary AAs (21.90 ± 1.76 and 8.58 ± 0.76% of protein), and tryptophan and cysteine were the least (1.15 ± 0.07 and 1.51 ± 0.15% of protein). Among amino acid groups, the highest and lowest contributions to the total protein intake were acidic and sulfuric AAs.

**Table 2:** Dietary intakes of participants of the Tehran Lipid and Glucose Study (2008-2011)

Variables	Total (n = 4,976)	Males (n = 2,230)	Females (n = 2,746)	P-value
Energy (kcal)	2,484 ± 916	2,582 ± 745	2,404 ± 1,028	<0.001
Carbohydrates (% of energy)	56.7 ± 6.1	58.0 ± 5.8	55.7 ± 6.2	<0.001
Total protein (% of energy)	13.3 ± 2.5	13.4 ± 2.1	13.3 ± 2.7	0.051
Total fat (% of energy)	28.9 ± 6.0	27.6 ± 5.5	29.9 ± 6.2	<0.001
Saturated fatty acid (% of energy)	9.9 ± 2.5	9.4 ± 2.6	10.3 ± 2.4	0.011
Fiber (g/1,000 kcal)	15.1 ± 5.2	15.1 ± 6.5	16.3 ± 19.3	0.002
**Amino acids**				
Tryptophan (% protein)	1.15 ± 0.07	1.16 ± 0.07	1.14 ± 0.07	<0.001
Isoleucine (% protein)	4.53 ± 0.21	4.51 ± 0.20	4.54 ± 0.22	<0.001
Lysine (% protein)	6.11 ± 0.67	6.00 ± 0.67	6.22 ± 0.66	<0.001
Cysteine (% protein)	1.51 ± 0.15	1.55 ± 0.15	1.48 ± 0.15	<0.001
Tyrosine (% protein)	3.53 ± 0.24	3.52 ± 0.23	3.55 ± 0.25	<0.001
Arginine (% protein)	5.29 ± 0.46	5.31 ± 0.43	5.27 ± 0.47	0.009
Alanine (% protein)	4.46 ± 0.25	4.46 ± 0.24	4.47 ± 0.25	0.100
Glutamic acid (% protein)	21.90 ± 1.79	22.21 ± 1.84	21.66 ± 1.71	<0.001
Threonine (% protein)	3.73 ± 0.17	3.70 ± 0.16	3.75 ± 0.17	<0.001
Leucine (% protein)	7.86 ± 0.39	7.85 ± 0.37	7.87 ± 0.41	0.130
Methionine (% protein)	2.16 ± 0.16	2.16 ± 0.15	2.17 ± 0.16	0.064
Phenylalanine (% protein)	4.70 ± 0.19	4.72 ± 0.18	4.68 ± 0.20	<0.001
Valine (% protein)	5.55 ± 0.36	5.53 ± 0.35	5.58 ± 0.38	<0.001
Histidine (% protein)	2.52 ± 0.10	2.52 ± 0.09	2.53 ± 0.10	<0.001
Aspartic acid (% protein)	8.58 ± 0.76	8.40 ± 0.69	8.72 ± 0.78	<0.001
Glycine (% protein)	3.80 ± 0.31	3.84 ± 0.30	3.76 ± 0.31	<0.001
Serine (% protein)	5.04 ± 0.28	5.03 ± 0.27	5.05 ± 0.28	0.088
Proline (% protein)	7.71 ± 0.81	7.80 ± 0.81	7.66 ± 0.81	<0.001
Lysine/Arginine Ratio	1.16 ± 0.17	1.13 ± 0.16	1.18 ± 0.17	<0.001
**Amino acid groups**				
Branched chain AAs (% protein)	17.95 ± 0.94	17.91 ± 0.90	18.03 ± 0.98	0.001
Aromatic AAs (% protein)	9.39 ± 0.38	9.40 ± 0.36	9.38 ± 0.40	0.077
Alkaline AAs (% protein)	13.94 ± 0.85	13.84 ± 0.85	14.03 ± 0.84	<0.001
Acidic AAs (% protein)	30.49 ± 1.42	30.61 ± 1.44	30.39 ± 1.39	<0.001
Sulfuric AAs (% protein)	3.68 ± 0.16	3.71 ± 0.14	3.65 ± 0.17	<0.001
Alcoholic AAs (% protein)	8.77 ± 0.37	8.74 ± 0.36	8.80 ± 0.39	<0.001

Data represented as mean ±SD or number and percent.

AAs which more supplied from animal food sources were lysine (68%), methionine (62%), tyrosine (59%), isoleucine (57%), threonine, valine, and leucine (57%), histidine (54%), alanine, and serine (53%). In contrast, some others were supplied more from plant food sources, including cysteine (61%), glutamic acid (58%), arginine (57%), tyrosine (55%), and glycine (54%) (Supplementary table). Dairy products, meats, fish, and eggs were the primary animal sources of AAs; bread, cereals & legumes, fruits & vegetables, and nuts formed the richest AA plant sources (Supplementary Figures 1 and 2, Supplementary Table).

Table 3 presents the association between each quartile increase in individual or AA groups’ dietary intakes with changes in body weight, WC, and BAI. Lysine, aspartic acid, and alkaline AAs were positively associated with weight changes after adjusting for potential confounders; whereas, cysteine, glutamic acid, and total sulfuric and acidic AAs were negatively related to weight changes. In the adjusted model, lysine, valine, and alkaline AAs showed a positive association with WC changes; however, there was a negative association between WC changes and dietary intakes of tryptophan, cysteine, glutamic acid, and total acidic AAs. Although several AAs have a significant relation with BAI changes in the crude linear regression model, only aromatic and acidic AAs showed a significant negative association with BAI changes in the adjusted model.

**Table 3:** Multiple linear regression analysis evaluating the association between single and groups of amino acids and anthropometric indices among adult participants the Tehran Lipid and Glucose Study

	Weight change		Waist circumference change		Body adiposity index change	
	Model 1*	Model 2†	Model 1*	Model 2†	Model 1*	Model 2†
	β (95% CI)	β (95% CI)	β (95% CI)	β (95% CI)	β (95% CI)	β* (95% CI)
**Amino acids**						
Tryptophan	-0.09 (-0.22, 0.03)	-0.11 (-0.24, 0.01)	**-0.18 (-0.33, -0.03)**	**-0.23 (-0.38, -0.07)**	**-0.06 (-0.13, -0.00)**	-0.02 (-0.08, 0.03)
Isoleucine	0.00 (-0.12, 0.13)	0.04 (-0.09, 0.18)	0.06 (-0.08, 0.21)	0.09 (-0.06, 0.25)	0.05 (-0.00, 0.12)	-0.00 (-0.07, 0.06)
Lysine	0.09 (-0.03, 0.22)	**0.16 (0.03, 0.30)**	0.13 (-0.01, 0.28)	**0.21 (0.05, 0.37)**	**0.09 (0.03, 0.15)**	0.02 (-0.03, 0.09)
Cysteine	0.00 (-0.12, 0.13)	**-0.16 (-0.30, -0.02)**	-0.05 (-0.20, 0.09)	**-0.16 (-0.32, -0.00)**	**-0.08 (-0.15, -0.02)**	-0.01 (-0.08, 0.04)
Tyrosine	0.06 (-0.06, 0.20)	0.07 (-0.05, 0.21)	0.08 (-0.06, 0.23)	0.10 (-0.05, 0.26)	0.04 (-0.02, 0.10)	-0.01 (-0.08, 0.04)
Arginine	0.10 (-0.03, 0.23)	0.01 (-0.11, 0.14)	0.01 (-0.13, 0.16)	-0.02 (-0.17, 0.12)	-0.01 (-0.07, 0.05)	-0.01 (-0.07, 0.05)
Alanine	0.05 (-0.07, 0.18)	0.05 (-0.08, 0.18)	0.08 (-0.06, 0.23)	0.10 (-0.04, 0.26)	0.04 (-0.02, 0.10)	0.01 (-0.04, 0.07)
Glutamic acid	**-0.21 (-0.34, -0.07)**	**-0.18 (-0.31, -0.05)**	-0.14 (-0.29, 0.00)	**-0.20 (-0.35, -0.04)**	**-0.10 (-0.17, -0.04)**	-0.04 (-0.10, 0.02)
Threonine	-0.00 (-0.14, 0.12)	0.09 (-0.04, 0.22)	0.03 (-0.12, 0.18)	0.09 (-0.06, 0.25)	0.05 (-0.00, 0.11)	-0.01 (-0.07, 0.05)
Leucine	0.02 (-0.11, 0.15)	0.01 (-0.12, 0.14)	0.09 (-0.05, 0.24)	0.09 (-0.06, 0.25)	0.03 (-0.03, 0.09)	-0.02 (-0.09, 0.04)
Methionine	0.04 (-0.08, 0.17)	0.03 (-0.10, 0.17)	0.14 (-0.00, 0.29)	0.17 (0.01, 0.34)	0.05 (-0.00, 0.12)	0.01 (-0.04, 0.08)
Phenylalanine	-0.10 (-0.23, 0.03)	-0.12 (-0.25, 0.00)	-0.01 (-0.16, 0.13)	-0.07 (-0.22, 0.08)	**-0.06 (-0.13, -0.00)**	-0.06 (-0.12, 0.00)
Valine	0.01 (-0.11, 0.14)	0.05 (-0.08, 0.18)	**0.15 (0.00, 0.30)**	**0.17 (0.02, 0.33)**	0.00 (-0.05, 0.07)	-0.04 (-0.10, 0.02)
Histidine	-0.08 (-0.21, 0.04)	0.02 (-0.10, 0.16)	0.04 (-0.10, 0.19)	0.09 (-0.06, 0.24)	0.02 (-0.03, 0.08)	-0.01 (-0.07, 0.05)
Aspartic acid	**0.18 (0.05, 0.31)**	**0.16 (0.03, 0.29)**	0.04 (-0.10, 0.20)	0.12 (-0.03, 0.27)	**0.09 (0.02, 0.15)**	0.00 (-0.06, 0.06)
Glycine	0.03 (-0.09, 0.17)	-0.05 (-0.18, 0.08)	0.00 (-0.14, 0.15)	-0.05 (-0.20, 0.10)	-0.02 (-0.09, 0.03)	0.01 (-0.04, 0.08)
Serine	-0.04 (-0.17, 0.09)	-0.01 (-0.14, 0.12)	0.02 (-0.12, 0.18)	0.02 (-0.13, 0.17)	-0.02 (-0.09, 0.03)	-0.05 (-0.12, 0.00)
Proline	**-0.15 (-0.28, -0.02)**	-0.10 (-0.23, 0.02)	-0.07 (-0.22, 0.07)	-0.10 (-0.25, 0.05)	**-0.07 (-0.13, -0.01)**	-0.03 (-0.09, 0.03)
Lysine/Arginine Ratio	0.01 (-0.11, 0.14)	0.10 (-0.03, 0.23)	0.07 (-0.07, 0.22)	0.15 (-.00, 0.31)	**0.08 (0.01, 0.14)**	0.01 (-0.04, 0.08)
**Amino acid groups**						
Branched chain AAs	0.03 (-0.09, 0.16)	0.06 (-0.07, 0.19)	0.11 (-0.04, 0.26)	0.12 (-0.03, 0.28)	0.02 (-0.03, 0.08)	-0.02 (-0.09, 0.03)
Aromatic AAs	-0.07 (-0.20, 0.06)	-0.10 (-0.23, 0.03)	-0.00 (-0.15, 0.14)	-0.05 (-0.20, 0.10)	-0.04 (-0.10, 0.02)	**-0.07 (-0.14, -0.01)**
Alkaline AAs	0.12 (-0.00, 0.26)	**0.15 (0.02, 0.28)**	0.14 (-0.00, 0.29)	**0.18 (0.03, 0.34)**	0.05 (-0.00, 0.12)	-0.00 (-0.06, 0.06)
Acidic AAs	**-0.19 (-0.33, -0.06)**	**-0.19 (-0.32, -0.06)**	**-0.19 (-0.35, -0.04)**	**-0.24 (-0.39, -0.08)**	**-0.11 (-0.17, -0.05)**	**-0.07 (-0.13, -0.00)**
Sulfuric AAs	0.02 (-0.11, 0.15)	**-0.17 (-0.30, -0.03)**	0.07 (-0.08, 0.22)	-0.02 (-0.18, 0.13)	-0.04 (-0.11, 0.01)	-0.02 (-0.08, 0.04)
Alcoholic AAs	-0.02 (-0.15, 0.10)	0.04 (-0.08, 0.18)	0.06 (-0.08, 0.21)	0.09 (-0.06, 0.25)	0.00 (-0.05, 0.07)	-0.04 (-0.11, 0.02)

Weight and waist circumference change values in follow-up time were entered as a dependent variable. Each quartile increase of dietary amino acids in the study’s baseline was entered for analysis as independent variables, and the β coefficient with 95% CI was reported.

* Model 1: Baseline values of weight, WC, and BAI were adjusted for weight change, waist circumference change, and body adiposity index change, respectively.

† Model 2: Analyses additionally adjusted for potential confounders: age, sex, physical activity, smoking (yes or no), daily intake of energy, total fat, and fiber intake.

Significant associations are in bold.

The association between AAs’ dietary intake as individuals or AA groups with the incidence of overweight & obesity and obesity-WC is presented in table 4. After 3-years of follow up 332 (21.1%) and 393 (13.5%) cases of overweight & obesity and obesity-WC incidence were ascertained, respectively (data not shown in table). The OR (95%CI) of overweight & obesity incidence per increasing each quartile of respective phenylalanine and Aromatic AAs was (0.87; 0.77-0.99, P value = 0.045) and (0.86; 0.75-0.97, P value = 0.022) in the final adjusted model for potential confounders. Other AAs showed no significant association with the incidence of overweight & obesity.

**Table 4:** Association between dietary intake of amino acids as an individual or amino acid groups and incidence of obesity following 3-years of study among participants of the Tehran Lipid and Glucose Study

	Overweight & obesity (BMI cutoff)*			Obesity (WC cutoff)†	
	Model 1‡	Model 2¶		Model 1‡	Model 2¶
	OR (95% CI)	OR (95% CI)¶		OR (95% CI)	OR* (95% CI)
**Amino acids**					
Tryptophan	**0.86 (0.76-0.97)**	0.88 (0.78-1.00)		**0.87 (0.78-0.96)**	**0.89 (0.80-0.99)**
Isoleucine	0.94 (0.83-1.06)	0.93 (0.81-1.07)		1.08 (0.97-1.19)	1.07 (0.96-1.20)
Lysine	1.08 (0.96-1.22)	1.06 (0.93-1.20)		**1.15 (1.04-1.28)**	**1.14 (1.02-1.28)**
Cysteine	0.92 (0.82-1.04)	0.92 (0.81-1.05)		0.93 (0.84 -1.03)	0.95 (0.85-1.07)
Tyrosine	0.96 (0.85-1.09)	0.94 (0.82-1.08)		1.04 (0.94-1.16)	1.03 (0.92-1.14)
Arginine	1.02 (0.91-1.15)	1.01 (0.89-1.15)		1.10 (0.99-1.22)	**1.12 (1.01-1.24)**
Alanine	1.01 (0.90-1.14)	1.00 (0.88-1.13)		**1.13 (1.02-1.26)**	**1.13 (1.01-1.25)**
Glutamic acid	**0.87 (0.77-0.98)**	0.92 (0.81-1.04)		**0.86 (0.78-0.96)**	**0.88 (0.79-0.98)**
Threonine	0.98 (0.86-1.11)	0.99 (0.87-1.13)		1.09 (0.99-1.21)	1.08 (0.97-1.21)
Leucine	0.95 (0.84-1.07)	0.93 (0.81-1.06)		1.05 (0.95-1.17)	1.04 (0.93-1.16)
Methionine	1.04 (0.92-1.17)	1.03 (0.89-1.18)		**1.12 (1.01-1.25)**	**1.13 (1.01-1.26)**
Phenylalanine	**0.86 (0.76-0.97)**	**0.87 (0.77-0.99)**		0.98 (0.88-1.09)	0.99 (0.89-1.10)
Valine	0.92 (0.81-1.04)	0.92 (0.80-1.05)		1.07 (0.96-1.18)	1.05 (0.95-1.17)
Histidine	1.00 (0.89-1.13)	1.03 (0.91-1.17)		1.09 (0.98-1.21)	1.09 (0.98-1.22)
Aspartic acid	1.08 (0.95-1.21)	1.01(0.89-1.15)		**1.17 (1.06-1.30)**	**1.14 (1.03-1.28)**
Glycine	1.05 (0.93-1.18)	1.04 (0.91-1.18)		1.03 (0.93-1.15)	1.06 (0.95-1.17)
Serine	**0.87 (0.76-0.98)**	0.88 (0.78-1.01)		0.98 (0.88-1.09)	0.97 (0.88-1.08)
Proline	0.92 (0.82-1.04)	0.96 (0.85-1.08)		**0.88 (0.79-0.98)**	**0.89 (0.80-0.99)**
Lysine/Arginine Ratio	1.05 (0.93-1.18)	1.04 (0.91-1.18)		1.05 (0.95-1.17)	1.03 (0.92-1.15)
**Amino acid groups**					
Branched chain AAs	0.94 (0.83-1.06)	0.93 (0.81-1.07)		1.06 (0.96-1.18)	1.05 (0.94-1.17)
Aromatic AAs	**0.86 (0.77-0.98)**	**0.86 (0.75-0.97)**		0.99 (0.89-1.10)	0.99 (0.88-1.09)
Alkaline AAs	1.05 (0.93-1.18)	1.02 (0.90-1.16)		**1.16 (1.05-1.29)**	**1.15 (1.04-1.29)**
Acidic AAs	0.89 (0.79-1.00)	0.92 (0.81-1.04)		**0.88 (0.79-0.97)**	**0.89 (0.80-0.99)**
Sulfuric AAs	0.98 (0.87-1.11)	0.94 (0.81-1.08)		1.07 (0.96-1.19)	1.10 (0.98-1.23)
Alcoholic AAs	**0.86 (0.76-0.97)**	0.88 (0.77-1.00)		1.03 (0.93-1.14)	1.02 (0.91-1.14)

Overweight & obesity or obesity incidence as a dichotomous variable was entered as a dependent variable. Each quartile increase of dietary amino acids in the study’s baseline was entered for analysis as independent variables and OR with 95% CI reported.

* Overweight and obesity were defined based on BMI ≥ 25.

† The waist circumference cutoff points for obesity were 99.5 cm for men and 94.25 cm for women.

‡ Model 1: Baseline values of BMI and WC were adjusted for overweight & obesity based on BMI and obesity based on WC cutoff, respectively.

¶ Model 2: Analyses additionally adjusted for potential confounders: age, sex, physical activity, smoking (yes or no), daily intake of energy, total fat, and fiber intake.

Significant associations are in bold.

Each quartile increasing the dietary lysine (OR: 1.14, 95% CI: 1.02-1.28, P value = 0.018), arginine (OR: 1.12, 95% CI: 1.01-1.24, P value = 0.029), alanine (OR: 1.13, 95% CI: 1.01-1.25, P value = 0.022), methionine (OR: 1.13, 95% CI: 1.01-1.26, P value = 0.033), aspartic acid (OR: 1.14, 95% CI: 1.03-1.28, P value = 0.012), and alkaline AAs (OR: 1.15, 95% CI: 1.04-1.29, P value = 0.007) related with greater risk of obesity-WC, while dietary intake of tryptophan (OR: 0.89, 95% CI: 0.80-0.99, P value = 0.035), glutamic acid (OR: 0.88, 95% CI: 0.79-0.98, P value = 0.028), proline (OR: 0.89, 95% CI: 0.80-0.99, P value = 0.036), and acidic AAs (OR: 0.89, 95% CI: 0.80-0.99, P value = 0.042) associated with lower risk of obesity-WC.

## DISCUSSION

This study investigated the association of individual and AA groups with 3-year changes in weight, WC, and BAI and the incidence of overweight & obesity and obesity-WC in a population-based cohort study of Tehrani adults. We designed two sets of cohort analyses and assessed all available anthropometric indices in our population study (height, weight, hip, and WC). To minimize data loss, we once investigated the potential role of AAs in changes of weight, WC, and BAI during follow-up regardless of the baseline body composition status of participants, and again we considered the subjects’ baseline body composition status and excluded those who were overweight and obese using two different cuts off for obesity. Then we analyzed the potential role of AAs in the incidence of overweight & obesity and obesity-WC. Indeed, we evaluated the AAs relationship with five types of anthropometric indices including weight changes, WC, and BAI, and also overweight & obesity and obesity-WC. Consequently, we sought similar findings and most repeated results that were as follows: protective association of acidic AAs (all indices except overweight & obesity), glutamic acid (3 indices of weight and WC changes and obesity-WC incident), and aromatic AAs (2 indices of BAI and overweight & obesity), cysteine and Sulfuric AAs (2 indices of weight and WC changes). Also, the direct relation of Alkaline AAs and lysine (3 indices of weight and WC changes and obesity-WC incident) and aspartic acid (2 heterogeneous indices including weight and obesity-WC). Our findings also indicated seven other AAs that each alone related to only one anthropometric index as follows: direct associations observed between alanine, arginine, and methionine with the incidence of obesity-WC and valine with WC changes, inverse relation between tryptophan and WC, proline and obesity-WC cutoff, and phenylalanine with overweight & obesity.

According to our knowledge, this was the first study investigating the association between AAs’ dietary intakes with future changes in weight, WC, and BAI. Okekunle and cols., in a cross-sectional study, found that higher intakes of 13 AAs including isoleucine, leucine, valine, lysine, cysteine, phenylalanine, tyrosine, threonine, histidine, aspartic acid, glutamic acid, proline, serine, and total AAs were inversely associated with obesity risk (13). Although our findings of cysteine, glutamic acid, phenylalanine, ad proline are consistent with the Okekunle study, we found a positive relationship of lysine, aspartic acid, and valine with anthropometric indices. Our study has several differences in terms of study design, statistical approaches, and adjusted variables, which could justify these different results. In addition, we expressed AAs as a percentage of total dietary protein, which can better be controlled for collinearity among AAs and other dietary variables, thus having less possibility for confounding the analyses compared to when AAs are expressed as grams per day or percentage of total energy (22).

Regarding the present study results, it seems that the highest reproducibility and reliability of our finding of AAs association with anthropometric indices is related to acidic AAs, then equally glutamic acid, alkaline AAs and lysine, and then equally aromatic and sulfuric AAs, cysteine, and aspartic acid. For the other 7 AAs mentioned above, which were related to anthropometric indices, we cannot refuse the probability of finding these results because of lack of repeated findings and significant differences between these AAs association with anthropometric indices in previous studies.

Acidic AAs (72% glutamic acid and 28% aspartic acid) contained 30.5% of total protein intake in our study and it was supplied more from plant sources (58% of glutamic acid and 51% of aspartic acid). It seems that the protective relation of acidic AAs is mostly related to its component, glutamic acid, which showed an inverse association with weight and WC changes and obesity-WC incident; otherwise, aspartic acid as another component of acidic AAs showed a positive relation with weight gain and overweight & obesity. Studies about glutamic acid and obesity-related factors are scarce. Only one previous cross-sectional study showed an inverse association between glutamic acid and obesity; however, animal studies showed that its supplementation suppressed food intake and reduces body fat and weight gain (23,24). Glutamic acid is the precursor of Gamma-aminobutyric acid (GABA), a chief inhibitory neurotransmitter that leads to higher food consumption when its receptors are inhibited. Furthermore, glutathione, an antioxidant molecule containing glutamate, cysteine, and glycine, reduced oxidative stress and the accumulation of reactive oxygen species, consequently, preventing inflammation and insulin resistance as risk factors of obesity (25).

In the present study, a positive association was found between dietary Alkaline AAs, particularly lysine with weight and WC changes, and the incidence of obesity-WC as well as arginine with the obesity-WC incident. Lysine is a ketogenic AA that is metabolized to acetyl COA, subsequently synthesizing body fats. Regarding the direct association of excess body weight and hyperlipidemia, our results support previous findings which indicated a direct relation between higher lysine and aspartic acid intake with an elevation of triglycerides and total cholesterol (26); or lysine contribution to body fat accumulation through hypercholesterolemic and hypertriglyceridemic effects (27).

We observed the inverse relationship between total Aromatic AAs with BIA changes and overweight & obesity incidents as well as aromatic AA components, including phenylalanine and tryptophan, with the incidence of overweight & obesity and WC changes. These two AAs previously showed protective relation with obesity, prediabetes, and WC (13,28); however, studies in this area are limited, and the mechanisms explaining these associations are unclear.

Sulfuric AAs, including cysteine and methionine, showed contradictory results in studies. In our study, Sulfuric AAs and cysteine were negatively associated with weight, and WC changes and methionine were related to the obesity-WC incident. However, in the Li and cols. study, Sulfuric AAs and cysteine significantly correlated with BMI and WC and were related to higher odds of obesity, and methionine showed any association with body composition indices (15). These conflicting results may be related to differences in study design, race, study sample size, analytical approach, and other unknown factors.

Some cross-sectional studies assessed the BCAAs-obesity relationship and showed that higher compared to lower intake of BCAAs was related with lower odds of obesity in Chinese, Japanese, and Brazilian populations (12,14,29,30). However, similar to our results, studies on the US and UK populations showed any significant association (29). It seems, besides differences in race, food habits, study design, etc., the food supply of BCAAs, despite similar intakes, may contribute to different associations of these AAs with chronic diseases. In Iranian and US populations, BCAAs are supplied more from dairy, cereals, and meats; however, the Japanese population is consumed more from cereals, fish, and shellfish (31).

The reasons underlying the conflicting results remain unclear. However, a hypothesis says that sources of protein may explain some of the inconsistencies. Previous studies have shown that dietary protein sources are related to subsequent weight and WC changes and risk of obesity. The animal protein showed a positive association with subsequent weight gain (10,32), whereas plant protein indicated an inverse association (33). Our results are consistent with this hypothesis so that most individual and AA groups such as alkaline AAs, lysine, methionine, valine, and alanine are positively related to weight gain, WC, and obesity supplied more from animal sources. In contrast, most AAs, including acidic AAs, glutamic acid, cysteine, tryptophan, and proline showed a negative relationship with anthropometric indices consumed more from plant sources or non-meat foods of animal sources. However, arginine and aspartic acid did not support this hypothesis.

The present study has considerable strengths. Compared to other cross-sectional studies on the association between AAs and anthropometric indices, to our knowledge, this is the first study that investigated the association between dietary AAs and anthropometric indices in the frame of a large prospective cohort study with a sufficient follow-up period to detect changes and incident case in anthropometric indices. In addition, we used various statistical methods and indices to find the most reliable results. Furthermore, trained dieticians collected dietary intakes using a valid and reliable FFQ for the estimation of dietary AAs. However, our study has some limitations, too; the present study is observational, and conclusions about causality cannot be drawn. Despite adjusting a wide range of variables, the confounding effect of some unknown and unmeasured residual confounders may have occurred.

In conclusion, higher dietary intakes of alkaline AAs, lysine, and aspartic acid may have adverse effects on anthropometric indices. In contrast, higher consumption of acidic AAs, glutamic acid, aromatic AAs, sulfuric AAs, and cysteine may negatively associate with body composition indices. Additionally, the present study observed direct associations for alanine, arginine, valine, methionine, the inverse association for tryptophan, proline, and phenylalanine with anthropometric markers.

These findings might warrant further researches in the form of a randomized controlled trial. In addition, we suggest applying more prospective studies in other populations for determining the beneficial and detrimental AAs concerning anthropometric indices.

## Figures and Tables

**Figure:**
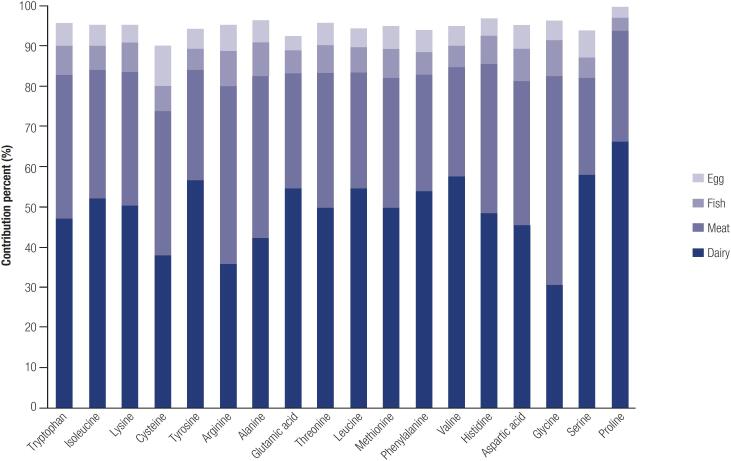
The main animal sources of dietary amino acids among participants of Tehran Lipid and Glucose Study (2009-2011)

**Figure:**
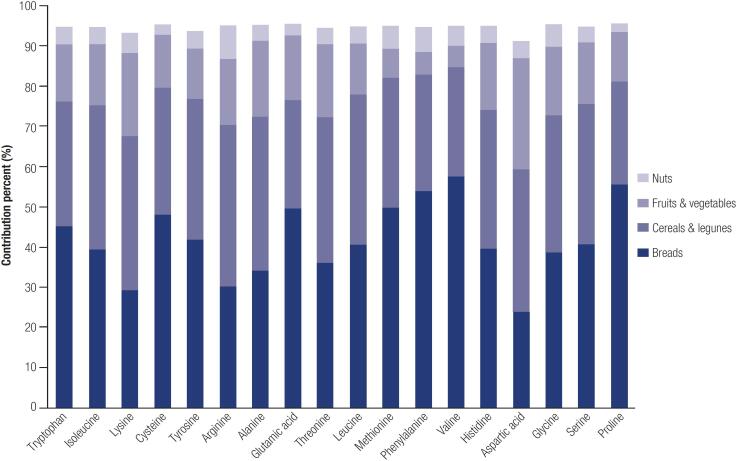
The main plant sources of dietary amino acids among participants of Tehran Lipid and Glucose Study (2009-2011)

## Supplementary Table.

**Table:** Main dietary sources of amino acids intake (mean % of total intake)

	Animal-Total (%)	Animal sources (100%)				Plant sources (100%)			
Amino acids		Dairy (%)	Meat (%)	Fish (%)	Egg (%)	Bread (%)	Cereals & legumes (%)	Fruits & vegetables (%)	Nuts (%)
Tryptophan	45.0	47.2	35.7	7.2	5.7	45.2	30.9	14.2	4.2
Isoleucine	57.0	52.1	32.0	6.0	5.2	39.5	35.6	15.0	4.3
Lysine	68.0	50.3	33.4	7.3	4.2	29.4	38.1	20.5	5.0
Cysteine	39.0	37.9	36.0	6.3	9.7	48.1	31.4	13.0	2.6
Tyrosine	59.0	56.9	27.1	5.5	4.8	41.9	34.8	12.3	4.4
Arginine	43.0	35.8	44.3	8.7	6.5	30.3	39.9	16.3	8.3
Alanine	53.0	42.2	40.2	8.3	5.7	34.2	38.0	18.8	4.0
Glutamic acid	42.0	54.6	28.6	5.7	3.6	49.6	26.7	16.2	2.8
Threonine	56.0	49.7	33.6	7.0	5.6	36.1	36.0	18.1	4.0
Leucine	56.0	54.3	29.2	6.2	4.8	40.6	37.1	12.7	4.2
Methionine	62.0	49.7	32.4	7.2	5.6	40.6	35.2	13.8	4.2
Phenylalanine	50.0	53.8	29.0	5.7	5.6	41.3	35.6	13.6	3.8
Valine	56.0	57.4	27.2	5.6	4.8	38.0	36.3	14.9	4.9
Histidine	54.0	48.3	37.1	7.2	4.3	39.7	34.3	16.5	4.2
Aspartic acid	49.0	45.5	35.8	8.1	5.9	24.1	35.1	27.6	4.3
Glycine	46.0	30.6	51.9	9.1	4.8	38.6	33.9	17.0	5.5
Serine	53.0	58.0	23.9	5.2	6.8	40.7	34.6	15.2	3.9
Proline	51.0	66.2	27.6	3.3	2.6	55.6	25.3	12.3	2.2

Data are presented as the mean intake of any amino acids as percent from total animal or percent from total plant sources.

&: and
